# Changes in the Expression of Biofilm-Associated Surface Proteins in* Staphylococcus aureus* Food-Environmental Isolates Subjected to Sublethal Concentrations of Disinfectants

**DOI:** 10.1155/2016/4034517

**Published:** 2016-10-27

**Authors:** Lenka Cincarova, Ondrej Polansky, Vladimir Babak, Pavel Kulich, Petr Kralik

**Affiliations:** ^1^Department of Food and Feed Safety, Veterinary Research Institute, Hudcova 70, Brno, Czech Republic; ^2^Department of Immunology, Veterinary Research Institute, Hudcova 70, Brno, Czech Republic; ^3^Department of Chemistry and Toxicology, Veterinary Research Institute, Brno, Czech Republic

## Abstract

Sublethal concentrations (sub-MICs) of certain disinfectants are no longer effective in removing biofilms from abiotic surfaces and can even promote the formation of biofilms. Bacterial cells can probably adapt to these low concentrations of disinfectants and defend themselves by way of biofilm formation. In this paper, we report on three* Staphylococcus aureus* biofilm formers (strong B+++, moderate B++, and weak B+) that were cultivated with sub-MICs of commonly used disinfectants, ethanol or chloramine T, and quantified using Syto9 green fluorogenic nucleic acid stain. We demonstrate that 1.25–2.5% ethanol and 2500 *μ*g/mL chloramine T significantly enhanced* S. aureus* biofilm formation. To visualize differences in biofilm compactness between* S. aureus* biofilms in control medium, 1.25% ethanol, or 2500 *μ*g/mL chloramine T, scanning electron microscopy was used. To describe changes in abundance of surface-exposed proteins in ethanol- or chloramine T-treated biofilms, surface proteins were prepared using a novel trypsin shaving approach and quantified after dimethyl labeling by LC-LTQ/Orbitrap MS. Our data show that some proteins with adhesive functions and others with cell maintenance functions and virulence factor EsxA were significantly upregulated by both treatments. In contrast, immunoglobulin-binding protein A was significantly downregulated for both disinfectants. Significant differences were observed in the effect of the two disinfectants on the expression of surface proteins including some adhesins, foldase protein PrsA, and two virulence factors.

## 1. Introduction

Staphylococcal food poisoning is considered to be one of the most common foodborne diseases worldwide [1]. Food contamination arises mainly because of inadequately sanitized food-processing equipment and the subsequent formation of biofilms on surfaces [2].* Staphylococcus aureus* together with* Salmonella* spp.,* Campylobacter* spp., enterohemorrhagic* Escherichia coli*, and* Listeria monocytogenes* are the major pathogens that are tested for in the meat industry. Gutiérrez et al., 2012, prepared 442 isolates from food contact surfaces in dairy, meat, or seafood environments and the presence of* S. aureus* was confirmed in 6.1% of samples. The biofilm form of bacteria, in comparison with its free-floating planktonic counterpart, is much more resistant to disinfectants, antibiotics, and phagocytosis [3–5], potentially leading to substantial economic losses and health problems [6]. It was reported that low concentrations (sub-MIC; sub-minimal inhibitory concentration) of residual disinfectants may even provide an opportunity for pathogens to adapt and grow.

A biofilm can be defined as a sessile community of bacterial cells that are embedded in a matrix of extracellular polymeric substance (EPS). Although exopolysaccharides are essential components of the biofilm matrix, recent studies revealed that bacterial surface-exposed proteins probably play a substantial role in biofilm development. Cucarella et al., 2001, studied* S. aureus* adherence and identified a gene, inserted in the SapIbov2 pathogenicity island, which encodes a surface-exposed protein named Bap (biofilm-associated protein) [7]. The Bap protein is the first of a group of surface-exposed proteins involved in biofilm development to be identified. The* Bap* gene has since been detected in many isolates of staphylococcal species, however, with only low incidence in human isolates. Nevertheless, staphylococcal strains can differ in their pathogenic strategies and may not be dependent on the presence of* Bap* [8]. Cramton et al., 1999, also showed that cell-cell adhesion during biofilm formation is probably mediated via the* ica* locus and, further, that deletion of the* ica* genes (*icaADBC*) eliminates the ability to produce polysaccharide intercellular adhesin (PIA) and to form a biofilm* in vitro* [9]. However, it is now recognized that the accumulation of staphylococci can also be promoted by surface proteins in an* ica*-independent manner (particularly relevant for MRSA strains). These proteins are biofilm-associated proteins Bap [7], ClfB [10], FnBPs [11], SasC [12], SasG [13], and protein A [14]. ClfB, FnBPs, and protein A are widely distributed among strains. When expressed at high levels on the cell surface, FnBPs, protein A, and SasC can promote biofilm formation. However, the mechanisms are not yet clear [11].

Staphylococci are nonmotile and nonspore forming facultative anaerobes.* S. aureus* possesses many adhesion proteins on its surface, but it is not known how they interact with each other to form stable connections with the substrate. Foster et al., 2014, suggested a classification of cell-wall anchored proteins (CWA) based on the presence of motifs that have been defined by structure-function analysis and listed the main group of CWA [15]. The most prevalent group is the Microbial Surface Component Recognizing Adhesive Matrix Molecule (MSCRAMM) family. Many of them are able to bind multiple ligands of the extracellular matrix (ECM) and thus possess extensive substrate plasticity [16]. Expression of surface-exposed proteins can be altered by cultivation conditions: some proteins are expressed mainly in the exponential growth phase [17] and others in the stationary-phase of growth [18].

Cleaning agents containing ethanol are commonly used as disinfectants in food-processing environments. Ethanol is the most popular antibacteriocidal agent, mainly due to its volatile and harmless character; however, alcohols lack sporicidal action and they inadequately penetrate protein-rich materials. For this reason, alcohols are not optimal as single-agent antiseptics for the disposal of biofilms. The bactericidal activity of alcohols is related to their ability to disrupt membrane structures or functions, inhibit protein synthesis [19], interfere with cell division [20], and impair steady-state growth [21]. They also promote variations in fatty acid composition, and alterations in membranes, intracellular pH, and membrane potential [22].

Chloramine T belongs to the group of chlorine-releasing agents (CRAs) and its mechanism of action is not fully known. Chloramine T is bactericidal as well as virucidal [23] and is used in the food industry as an antimicrobial agent [24]. It is commonly used to manage biofilm growth [25]. Growth of a* S. aureus* biofilm can also be enhanced by some processing methods encountered in the food industry, such as suboptimal temperatures or a combination of salt and glucose [26].

The aim of this study was to determine effective concentrations of commonly used food industry disinfectants that can induce biofilm formation and to describe changes in the abundance of surface-exposed proteins during biofilm formation using enzymatic cell surface shaving, dimethyl labeling, and LC-LTQ/Orbitrap analysis.

Enzymatic shaving of intact bacterial cells is a novel, rapid method for identification of surface-exposed peptide epitopes and can be used for protein sorting without the use of, for example, gel separation (2-DE) or 2D-LC coupled with MS/MS [27, 28]. The cell-wall of Gram-positive bacteria is permeable to proteins of approximately 50 kDA and thus trypsin (23 kDa) can diffuse through the cell-wall. Surface-exposed proteins can be integral membrane proteins, lipoproteins, or cell-wall-associated proteins. Cell shaving can result in contamination with cytosolic factors and, thus, the best results have been achieved in Gram-positive bacteria whose thick peptidoglycan cell-walls are more resistant to spontaneous lysis in solution [28]. Tjalsma et al., 2008, also tested trypsin-beads, which are unable to penetrate the bacterial cell-wall and thus probably ensure genuine surface-exposed localization [27]. The trypsin shaving approach has been mainly used thus far in combination with MS analysis for identification of potential vaccine candidates in pathogens such as Streptococcus pyogenes or Bacillus subtilis [29, 30], for characterization of* S. aureus* surfactome interactions with host plasma proteins [31], and for characterization of* S. aureus* adhesins or other surface proteins with adhesive functions [32].

## 2. Materials and Methods

### 2.1. Biofilm Cultivation

*Staphylococcus aureus* samples were collected from food contact equipment in meat-processing plants. Sampling was carried out in 2 visits. Samples were taken aseptically from a surface covering approximately 100 cm^2^ by using a sterile sampling sponge moistened with LPT Neutralizing Broth and then transported at 4°C to the laboratory for immediate processing. Firstly, the strength of biofilm formation was determined according to Stepanović et al., 2007, [33] and three biofilm forming* S. aureus* isolates (B+++ strong biofilm former, internal collection number: 1275; B++ moderate biofilm former, 1863; B+ weak biofilm former, 1053; all three isolates were collected from knives used in slaughtering) were chosen for assessing the effect of disinfectants on biofilm growth. The* S. aureus* biofilm was grown in TSB (Oxoid Ltd., Hampshire, England) supplemented with 1% NaCl (Penta, Chrudim, Czech Republic) and 1% D-Glucose (Penta, Chrudim, Czech Republic). The* S. aureus* biofilms were cultivated in 96-well polystyrene Nongrowth Enhanced U-Bottomed Tissue Culture Plates (Falcon, NY, USA) with disinfectants (ethanol or chloramine T), which were diluted in supplemented TSB to their respective concentrations (Table 1). A* S. aureus* biofilm cultivated in supplemented TSB without disinfectants was considered as the control. To prepare biofilms, a* S. aureus* inoculum (5 h cultivation in shaking water bath at 37°C) was grown to 1.5 × 10^9^ cfu/mL and then diluted 1 : 100 in TSB and dispensed into 200 *μ*L aliquots in 96-well polystyrene Tissue Culture Plates for Syto9 quantification, or into 10 mL aliquots in 6-well polystyrene Flat-Bottomed Nongrowth Enhanced Tissue Culture Plates (Falcon) for surface-exposed protein extraction. Bacteria were incubated in their respective media (control or medium with disinfecting agents) in an incubator (Sanyo, Tokyo, Japan) at 37°C for 48 h. Media were changed after 24 h of cultivation, and biofilms were cultivated for 48 h.

### 2.2. Biofilm Quantification

*S. aureus* biofilms in 96-well Tissue Culture Plates were quantified after 48 h of growth using Syto9 Green Fluorescent Nucleic Acid Stain (Life Technologies, Eugen, Oregon, USA). After the medium was discarded, biofilms were washed with 200 *μ*L phosphate-buffered saline (PBS; pH = 7.2) at room temperature for 15 min at 250 rpm in a TS-100 thermoshaker (BioSan, Michigan, USA). Syto9 was diluted in PBS (1 : 3600). PBS and diluted Syto9 solution (100 *μ*L of each) were added consecutively per well to the washed biofilms. After 1 h of incubation in a thermoshaker at 37°C at 250 rpm in the dark, fluorescence was measured using a Synergy H1 Hybrid Reader (BioTek, Vermont, USA) (excitation: 478 nm, emission: 510 nm, and gain: 60%).

### 2.3. Scanning Electron Microscopy (SEM)

For SEM,* S. aureus* biofilm former B+ (1053) was grown on plastic cover slips (Falcon) for 48 h as described above. After biofilm cultivation slips were replaced, they were washed three times in PBS, fixed in 3% Millonig phosphate-buffered gluteraldehyde 3x for 10 min (Serva, Germany), postfixed in 2% Millonig osmium tetroxide buffered solution for 1 hour (Serva, Germany), and then washed 3x for 10 min in Millonig phosphate buffer. The samples were subsequently dehydrated in increasing concentrations of acetone (50, 70, 90, and 100%), every step for 20 min, and dried in hexamethyldisilazane (Sigma-Aldrich, Czech Republic) for 3 h in a hood at RT. Then, the samples were placed on carbon tabs attached to an aluminium holder and coated with platinum/palladium (Cressington sputter coater 208 HR, UK). The structure and interaction of biofilm cells were observed under a Hitachi SU 8010 scanning electron microscope (Hitachi High Technologies, Japan) at a magnification of 6000x (at 15 kV, wd 10.9 mm).

### 2.4. Cell Surface-Exposed Protein Extraction

For the extraction of surface-exposed proteins,* S. aureus* biofilms were cultivated in polystyrene 6-Well Clear Flat Bottom TC-Treated Multiwell Cell Culture Plates (TPP, Trasadingen, Switzerland) and their inoculum in 50 mL tubes (TPP, Trasadingen, Switzerland). The inoculum was used as a control (planktonic cells). The fraction of surface-exposed proteins was prepared by trypsin shaving according to Tjalsma et al., 2008 [27], with minor modifications. Briefly, after medium removal, biofilm cells were resuspended by repeated pipetting in PBS (pH = 7.2), washed twice in PBS, and centrifuged at 14000 ×g for 10 min. Washed biofilm cells were resuspended in 2 mL PBS. The cell number of washed biofilm cells and the inoculum were determined using qPCR. Equal amounts of biofilm cells and inoculum were incubated in a thermoshaker with trypsin to a final concentration of 1 *μ*g/mL at 37°C for 1 h at 550 rpm. After digestion, cells were centrifuged at 14000 ×g for 5 min and supernatants containing shaved proteins were stored. The concentration of “shaved protein” extracts was determined spectrophotometrically (A280) using a NanoDrop™ 2000/2000c (Thermo Scientific, Wilmington, USA). Three independent cultivations and subsequent extractions were carried out. Extracts were reduced, alkylated, and trypsinized prior to mass spectral analysis according to Wiśniewski et al., 2009 [34].

### 2.5. Protein Labeling for Quantitative Analysis

Protein quantification was based on multiplexed peptide stable isotope dimethyl labeling [35]. Samples (tryptic peptides) were dissolved in 100 mM triethylammonium bicarbonate (TEAB) and 4% formaldehyde CH_2_O (“light”). Deuterated formaldehyde CD_2_O (“intermediate”) or formaldehyde ^13^CH_2_O (“heavy”) were added followed by 4% sodium cyanoborohydride NaBH_3_CN (“light, intermediate”) or sodium cyanoborodeuteride NaBD_3_CN (“heavy”). The mixtures were incubated for 45 min at room temperature and quenched with 1% NH_3_. After addition of 8 *μ*M formic acid, 3 differentially labeled samples were pooled and desalted using Empore™ C18-SD 4 mm/1 mL SPE cartridges (Supelco, Bellefonte, Pennsylvania). Treated biofilm extracts were labeled “heavy,” the treated planktonic cell extracts were labeled “intermediate,” and inoculum extracts were labeled “light.” Labeled extracts were combined as follows: treated biofilm compared to inoculum and treated planktonic cells compared to inoculum. The surfactome of treated biofilm or treated planktonic cells and the inoculum were compared for identification of proteins whose abundance increased in response to cultivation of biofilms with disinfectants. Samples were analyzed using LC-LTQ/Orbitrap hybrid MS (Thermo Scientific, San Jose, California).

### 2.6. LC-MS/MS Analysis

LC-MS/MS analysis of tryptic peptides was performed on a Dionex UltiMate 3000 RSLC liquid chromatograph connected to a LTQ-Orbitrap Velos Pro hybrid mass spectrometer (Thermo Scientific). For each analysis, 5 *μ*g of peptide sample was used. Samples were separated on EASY Spray C18 columns (length 50 cm, ID 75 *μ*m, and particles 3 *μ*m) at a flow rate of 200 nL/min and a gradient of 1 hour. The mass spectrometer was operated in MS spectra data-dependent mode (Orbitrap analyzer, 30 000 FWHM resolution, mass range 390–1700* m*/*z*). The ten most abundant peptides were isolated and fragmented using collision-induced dissociation (CID) (normalized collision energy 35) followed by MS/MS scan (LTQ analyzer). Dynamic exclusion was enabled (30 s duration).

### 2.7. Data Analysis

Raw LC-MS/MS data were analyzed using Proteome Discoverer (v1.4). Tandem mass spectra identification was performed employing the SEQUEST algorithm. For each search, precursor and fragment mass tolerances were 10 ppm and 0.6 Da, respectively. Cysteine carbamidomethylation was set as a fixed modification; methionine oxidation was set as a dynamic modification. Only peptides with false discovery rates of ≤5% were considered.

### 2.8. Statistical Analysis

Data analysis was performed using the statistical software Statistica 9.1 (StatSoft, Inc., Tulsa, OK, USA) and GraphPad Prism 5.04 (GraphPad Software, Inc., San Diego, CA, USA). Data regarding the fluorescent quantification of biofilms using Syto9 labeling were analyzed by one-factor ANOVA followed by Dunnett's* post hoc* test (treatments versus control).

Evaluation of mass spectrometry quantification data was performed in such a way that medians of folds H/L (“heavy”/“light”) and M/L (“intermediate”/“light”) of selected proteins for all disinfectants were compared with the value 1.0 using the Wilcoxon signed rank test with null hypothesis: Median = 1.0.

## 3. Results

### 3.1. Syto9 Biofilm Quantification

**Figure 1:**
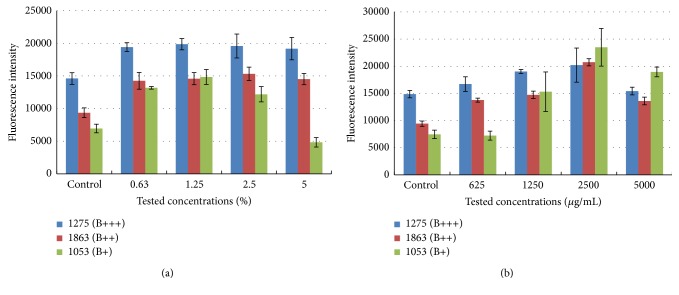
Syto9 quantification of 3 biofilm forming isolates of* S. aureus *treated with different concentrations of ethanol or chloramine T. Strong biofilm former (B+++), moderate biofilm former (B++), and weak biofilm former (B+) were treated with increasing concentrations of ethanol (a) or chloramine T (b) and biofilm quantity was determined by Syto9 labeling. Graphs show biofilm levels in samples cultivated with disinfectants versus controls (samples cultivated without disinfectants). Columns represent mean values of fluorescence and vertical bars represent 95% confidence intervals regarding the means.

Three chosen isolates of* S. aureus* (B+++ strong biofilm former, internal collection number: 1275; B++ moderate biofilm former, 1863; B+ weak biofilm former, 1053) were cultivated statically in 96-well plates for biofilm with varying concentrations of ethanol or chloramine T (treated cells) or without disinfectant (control cells). After 48 h of cultivation biofilms were quantified using the Syto9 green fluorogenic nucleic acid stain. Syto9 diffuses passively through cellular membranes and binds to DNA. As DNA also forms a substantial part of the extracellular matrix, this dye stains intracellular DNA as well as DNA in the extracellular matrix and thus provides information about total biofilm biomass regardless of whether the cells are alive or dead [35]. To determine sub-MICs of chosen disinfectants that promote biofilm formation, quantities of treated and control biofilms were compared. Statistical analysis revealed that biofilm formation by the biofilm formers was increased after application of disinfection reagents. For isolate B+++ (1275), ethanol, at concentrations from 0.63 to 5% (v/v), approximately equally promoted biofilm formation. Biofilms in treated samples were increased approximately 0.75x compared to the control. Similarly, for isolate B++ (1863), biofilm formation was increased approximately equally (0.66x) to all concentrations of ethanol tested. For the weak biofilm former B+ (1053) the biofilm grew progressively with increasing ethanol concentrations (from 0.63% to 1.25%) and reached a maximum at 1.25% ethanol; biofilm formation then decreased with further increase in ethanol concentration (5% ethanol). With chloramine T treatment, statistically significant biofilm formation by the strong biofilm former B+++ (1275) occurred only at concentrations of 1250 and 2500 *μ*g/mL. At 5000 *μ*g/mL no biofilm formation occurred. Biofilm formation by the moderate biofilm forming isolate B++ (1863) gradually increased with increasing concentrations of chloramine T, and maximum formation was observed at 2500 *μ*g/mL; after application of 5000 *μ*g/mL of chloramine T, biofilm formation decreased. Treatment of the weak biofilm producer B+ (1053) with 623 *μ*g/mL chloramine T did not lead to biofilm formation but this was significantly increased after treatment with 1250, 2500, and 5000 *μ*g/mL chloramine T. Maximum formation of biofilm was measured after treatment with 2500 *μ*g/mL chloramine T (*P* > 0.01; ANOVA, Dunnett's test; Figure 1).

### 3.2. Enzymatic Extraction of Surface Proteins (Trypsin “Shaving”)

The surface proteome was analyzed on* S. aureus* isolate 1053, in which the largest increase in biofilm formation was measured by Syto9 labeling (3.2x with 1.25% ethanol and 2.2x with 2500 *μ*g/mL chloramine T). The biofilm of* S. aureus* isolate 1053 was cultivated for 48 h in 6-well plates with a concentration of ethanol or chloramine T that was observed to promote biofilm formation (1.25% ethanol and 2500 *μ*g/mL chloramine T). Surface-exposed proteins were extracted from biofilms treated with chloramine T and from the inoculum using the trypsin shaving approach. The “harvest” from trypsin shaving was 0.75 mg of cell surface-exposed-protein extract from 1 × 10^9^ nontreated biofilm cells, 0.80 mg from treated biofilm cells, and 0.38 mg of cell surface-exposed protein extract from 1 × 10^9^ inoculum cells.

### 3.3. Mass Spectrometric Analysis

Extracts from 1.25% ethanol- and 2500 *μ*g/mL chloramine T-treated cells were examined by mass spectrometry and many unique proteins (1162 and 1321, resp.) were identified. Of these, 92 and 128, respectively, of the identified proteins were located in the membrane or cell surface or had an extracellular location.

Our data shows that 6 groups of proteins showed significant up- or downregulation in treated biofilm forming cells compared to the inoculum (Table 2). The following groups of proteins were identified: (1) adhesin proteins involved in surface adherence; (2) proteins involved in cell-wall synthesis and organization; (3) cell maintenance proteins; (4) nascent transmembrane protein transporters; (5) uncharacterized proteins; (6) virulence factors.

Several proteins were found to be significantly upregulated after treatment: from the adhesins: clumping factor A and extracellular adherence protein Eap; from cell maintenance proteins: large-conductance mechanosensitive channel, uncharacterized lipoprotein SAS2259, and virulence factor EsxA. Immunoglobulin-binding protein A was significantly downregulated with both disinfectants. A statistically significant difference in the effect of tested disinfectants on the expression of surface proteins was measured for adhesins: fibronectin-binding protein A (chloramine T, upregulation; ethanol, downregulation) and iron-regulated surface determinant protein A (ethanol, upregulation; chloramine T, downregulation); nascent transmembrane protein transporters: foldase protein PrsA (ethanol, upregulation; chloramine T, downregulation) and UPF0478 protein SA1560 (chloramine T, upregulation; ethanol, downregulation), and virulence factors: serine-aspartate repeat-containing protein C and staphylococcal secretory antigen ssaA2 (ethanol, upregulation; chloramine T, downregulation).

Significant upregulation of the following proteins was observed only in ethanol-treated biofilm cells compared to the inoculum: clumping factor B, immunoglobulin-binding protein sbi, and virulence factors penicillin-binding protein 1 and phospholipase C. Downregulation was observed for proteins involved in cell-wall synthesis and lipoteichoic acid synthase. Similarly, statistically significant differences were demonstrated only in chloramine T-treatment for the following proteins: from adhesins: fibrinogen-binding protein A (upregulation); from cell-wall synthesis and organization: N-acetylmuramoyl-L-alanine amidase sle1 and probable transglycosylase SceD (downregulation), and from cell maintenance proteins: cold shock protein CspA (downregulation).

### 3.4. Visualization of* S. aureus* Control and Treated Biofilms by Scanning Electron Microscopy

**Figure 2:**
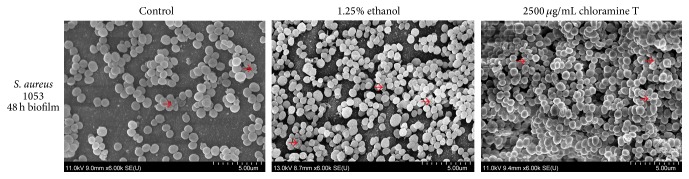
Representative SEM images of 48 h biofilm formed by B+ (1053) isolate in medium (control), 1.25% ethanol, or 2500 *μ*g/mL chloramine T. Arrows: extracellular matrix.

Control and 48 h biofilms treated with disinfectants differed not only in quantity, as determined by Syto9 labeling, but also in compactness; this, however, was not clearly captured by Syto9 labeling. For this reason we used scanning electron microscopy (SEM) to visualize the weak biofilm former B+ (1053), which was then used for MS analysis of the surface proteome (Figure 2). It was clearly seen from representative SEM images that biofilms formed after treatment with 1.25% ethanol or 2500 *μ*g/mL chloramine T were more compact in comparison with the control biofilm. In addition, the 2500 *μ*g/mL chloramine T-treated biofilm appeared to be more compact than the biofilm treated with 1.25% ethanol.

## 4. Discussion

It has been reported that sublethal concentrations (sub-MICs) of certain disinfectants are no longer effective in removing biofilms from abiotic surfaces and can even promote the formation of biofilms. Tolerance of bacterial biofilms to disinfectants increases the risk of cross-contamination of food. Bacterial cells probably react to the presence of disinfectants and defend themselves by way of biofilm formation [36]. As disinfectants diffuse through the biofilm matrix their concentration is lowered and bacterial cells can adapt. For example, biofilm formation of* S. epidermidis* exposed to benzalkonium chloride at 1/16, 1/18, and 1/32 of the MIC was increased from 11.4% to 22.5% without any significant effect on planktonic growth [37].

Our data showed that ethanol and chloramine T, at sub-MICs, are each capable of promoting biofilm formation by* S. aureus*. Different isolates of* S. aureus* from meat-processing environments were tested for biofilm formation according to the methods of Stepanović et al., 2007 [33]. One representative was a strong biofilm former (B+++), one was a moderate biofilm former (B++), and one was a weak biofilm former. These were chosen to quantify the effects of disinfectants on biofilm formation. The* S. aureus* isolates were cultivated in 96-well plates with different sublethal concentrations of ethanol, or chloramine T, and biofilm formation was quantified using Syto9 Green Fluorescent Nucleic Acid Stain. After application of 1.25% ethanol or 2500 *μ*g/mL chloramine T, not only was there an increase in biofilm formation, as depicted in Figure 1, but also there were changes in the quality of the biofilm compared to the control (the biofilms were firmer and more symmetrically proportioned after application of disinfectants). This qualitative aspect of biofilm formation cannot be fully captured by Syto9 staining, which is why visualization of treated and control biofilms was carried out by scanning electron microscopy (Figure 2).

Statistical analysis showed that 0.63–5% ethanol in case of strong and moderate biofilm formers and 0.63–2.5% ethanol in case of weak biofilm former significantly promoted biofilm formation. The maximum biofilm formation was observed for 2.5% ethanol for moderate and 1.25% ethanol for weak biofilm forming isolates. This finding is in agreement with previous studies [36] where elevated biofilm formation was observed after application of 1-2% ethanol. Ethanol, at 2.4% v/v, enhanced the expression of a number of biofilm-promoting genes in* S. aureus* [38]. It was also demonstrated that application of other alcohols (ethanol, methanol, isopropanol, isoamyl alcohol, and n-butanol) to preformed* S. aureus* biofilms enhanced biofilm growth [39].

The effect of sub-MICs of chloramine T on biofilm formation has not previously been tested. Our data showed a statistically significant increase in biofilm formation in response to 1250 and 2500 *μ*g/mL chloramine T in the strong isolate, 623–5000 *μ*g/mL in the moderate isolate, and 1250–5000 *μ*g/mL in the weak biofilm forming isolate compared to the control (Figure 1). Maximum biofilm formation was observed in all three isolates with 2500 *μ*g/mL of chloramine T.

It was reported that formation of biofilms can be enhanced not only by chemicals, but also by other stress conditions such as temperature [40, 41] or pH [42]. Ciccio et al., 2014, observed that 38 out of 67 tested* S. aureus* strains (57%) grew at 37°C on polystyrene or stainless steel, while, in comparison, only one strain grew at 12°C. They also observed that cell surface hydrophobicity levels increased with temperature.

Ethanol or chloramine T treatments of* S. aureus* biofilms were further analyzed to describe changes in the abundance of surface-exposed proteins after treatment. Enzymatic cell surface shaving, dimethyl labeling, and LC-LTQ/Orbitrap analysis were used to describe changes in the abundance of surface-exposed proteins in treated biofilms and the inoculum. To reduce the number of false positives and to correct for experimental variations, only those proteins with at least two unique peptides in three triplicate experiments were considered significant.

Our data shows that ethanol as well as chloramine T-treated* S. aureus* biofilm cells expressed higher levels of proteins associated with cell attachment than control cells. The observation that biofilm-producing cells overexpress adhesins compared to their planktonic counterparts is in agreement with other studies [43–45]. These proteins belong to the MSCRAMM group of surface-exposed proteins, but their biological importance and their roles in adhesion and virulence of* S. aureus* are not completely known. MSCRAMMs promote adhesion of* S. aureus* to the extracellular matrix, to the surface of host cells, and to biomaterial surfaces that are conditioned, for example, by the deposition of plasma proteins. Four* S. aureus* surface-exposed proteins, clumping factor A (ClfA), fibronectin-binding proteins A and B (FnBPA and FnBPB), and enolase, were found as the main factors involved in the adherence of* S. aureus* to polyurethane membranes of ventricular assist devices [46]. As fibronectin is present on epithelial and endothelial surfaces and is also part of blood clots, fibronectin-binding proteins (Fnbp A/B) and clumping proteins (Clp A and B) help* S. aureus* to invade these tissues [3]. Enolase was identified as a 52 kDa surface receptor of laminins [47] and, thus, may play a critical role in the pathogenesis of* S. aureus* by allowing its adherence to the laminin-containing extracellular matrix. Surface-exposed proteins directly or indirectly interact with integrins and promote the invasion of nonphagocytic host cells. Intercellular bacteria can cause host cell apoptosis or they can enter a nondisruptive semidormant state (“small colony variants”). These surface-exposed proteins probably also play a role in the accumulation of* S. aureus* cells during biofilm formation [11]. Differences between chloramine T and ethanol treatments were recorded for these proteins with adherence function: enolase, fibronectin-binding protein A, and iron-regulated surface determinant protein A (Table 2). The biofilm/inoculum ratios for enolase were approximately 1 for both treatments, which probably means that chloramine T does not lead to upregulation of enolase or ethanol to its downregulation. It is possible that inoculum cells express similar levels of enolase as biofilm cells in order to adhere. Our data show that chloramine T leads to upregulation of fibronectin-binding protein A (fib) and ethanol to its downregulation, despite the fact that fibrinogen-binding protein (fnb) was upregulated after both treatments. The difference between these two proteins is in their substrate plasticity. While fib binds preferentially to fibronectin, fnb protein binds to multiple substrates (Table 3). In contrast, iron-regulated surface determinant protein A (isdA) was downregulated after chloramine T-treatment and upregulated after ethanol treatment. IsdA protein also binds multiple ligands, for example, fibronectin, or contributes to bacterial cell adherence (Table 3). It is questionable whether these data suggest a disinfectant-specific response of* S. aureus*. The remaining identified proteins, on the contrary, might suggest a general stress response. This question would be better answered by a detailed analysis of whole cell extracts and confirmed using RT-PCR transcriptome analysis.

According to our data, proteins involved in cell-wall synthesis were shown to be predominantly downregulated in treated biofilm cells compared to the inoculum. This might have been due to differences in the growth phase between biofilm and inoculum cells: whilst, after 16 h of cultivation, inoculum cells should be in the early stationary-phase of growth, still multiplying and growing, biofilm cells are probably in the late stationary-phase of biofilm formation and differentiation, in which mainly proteins of the extracellular matrix are expressed.

The large-conductance mechanosensitive channel was upregulated after treatment with both disinfectants (Table 2). Probably this protein might participate in regulation of osmotic pressure induced by the presence of chloramine T or ethanol (Table 3). Foldase protein PrsA that participates in transport of secreted proteins through membranes was determined to be downregulated after chloramine T treatment and upregulated after ethanol treatment. Its upregulation may be associated with the fact that ethanol might disrupt the cell membrane or with another metabolic response to ethanol.

Virulence factors of* S. aureus*, such as phospholipase C, iron-regulated surface determinant protein A, staphylococcal secretory antigen ssaA2, and virulence factor EsxA, were also detected. Only virulence factor EsxA was found to be upregulated in the treated biofilm. This is in agreement with the claim that planktonic cells are generally more virulent than their biofilm counterparts [44]. The functions of these proteins are listed in Table 3. Perhaps the most striking differences between the chloramine T and ethanol treatments were measured for two virulence factors of* S. aureus*: serine-aspartate repeat-containing protein C (SdrC) and staphylococcal secretory antigen (ssaA2), which were both upregulated after treatment with ethanol and downregulated by chloramine T treatment. SdrC is a cell surface-associated protein that possibly mediates interactions of* S. aureus* with components of the extracellular matrix of higher eukaryotes. This protein contains the C termini LPXTG motifs and hydrophobic amino acid segments and thus is a characteristic member of surface proteins covalently anchored to peptidoglycan. Staphylococcal secretory antigen ssaA2 is an immunogenic protein of unknown function. It was also observed in other studies that ethanol increased the level of genes considered necessary for production and viability of the biofilm. These included icaAD, sdrDE, pyr, and ure [38]. Generally, exposure to ethanol increases pathogenic traits and induces oxidative-stress responses. This effect of ethanol might be related to the upregulation of sdrC and ssaA2 virulence factors.

The last group of proteins consisted of multiple uncharacterized proteins that could play an important role in biofilm development. Uncharacterized proteins that are upregulated in the biofilm are probably components of metabolic or physiological pathways of biofilm formation and differentiation. Some of these uncharacterized proteins might be stress response factors that could be expressed in response to the presence of disinfectants.

Expression of cell-wall associated proteins in this study, as well as in many other studies, was determined after cultivation in bacterial growth medium. However, when* S. aureus* contaminates, for example, a working table or knives in a food-processing environment, or infects a wound on human skin, the bacterial growth conditions will be quite different from those in medium* in vitro*, and this may affect the expression of surface-exposed proteins. Variable levels of single proteins might also be partly due to biological variation. After binding to the surface, biofilm cells usually become multilayered and differentiated. Growth conditions (supply of oxygen and nutrients) vary greatly among the various layers; this can promote differential growth and physiology and should also result in differences in protein expression.

Analysis of proteomic differences between biofilm and planktonic forms of different bacterial species is currently the subject of much research [48–50]. There are a number of reports on the expression of MSCRAMM adhesins, using one or two basic approaches: studies of surfactome expression at the transcriptome level or studies of surfactome expression at the proteome level. For the first approach, DNA microarray analysis that enables the simultaneous determination of the total transcriptional response is mainly applied [38, 45]. A disadvantage of this approach is that the level of mRNA can differ from the final level of its corresponding protein. For the second, combination of 2D-gel separation and mass spectrometry is generally employed [44, 50–53], or flow cytometry [54]. Enzymatic shaving is a novel and appropriate approach for surface-exposed protein extraction. It is a very simple and fast method and extracts obtained using enzymatic shaving contain minimal levels of cytoplasmic contaminants that could obscure minor amounts of surface-exposed proteins. The simple mixture of dimethyl-labeled samples is also an advantage for mass spectrometric analysis [28].

The major task for the future is to find more effective solutions for biofilm-associated contamination. Bacterial cells are able to adapt to low concentrations of disinfecting substances and form biofilm barriers. During the first step of biofilm formation, adhesive molecules are mainly expressed. They are one of the basic contributors to the survival, pathogenicity, and virulence of bacteria such as* S. aureus* and thus might represent markers for a molecule-targeted approach for the eradication of contaminating biofilms. Surface-exposed proteins are also currently being evaluated as potential antigens in vaccines [55, 56]. These topics require further investigation.

## 5. Conclusion

In the present work we have demonstrated that treatment of* S. aureus* isolates from a meat-processing environment with 1.25–2.5% ethanol or 2500 *μ*g/mL chloramine T enhanced biofilm formation as determined by Syto9 labeling. The change in compactness of the biofilm after treatment with ethanol or chloramine T was visualized by scanning electron microscopy. Further we demonstrated that trypsin shaving in combination with dimethyl labeling and high-resolution LC-MS/MS analysis serves as a rapid and valuable tool for studying changes in abundance of surface-exposed proteins connected with bacterial biofilm formation. Biofilm cell treated with 1.25% ethanol or 2500 *μ*g/mL chloramine T exhibited elevated expression of proteins that are involved in adhesion and sessile growth of* S. aureus*. The overall control of surface proteins appears to be more or less similar after administration of ethanol or chloramine T. The main differences were in regulation of some adhesins (fibronectin-binding protein A, iron-regulated surface determinant protein A), transport protein foldase protein PrsA, and virulence factors (serine-aspartate repeat-containing protein C; staphylococcal secretory antigen ssaA2). This work confirms results of previous studies where, using classical microbiological methods, some sub-MICs of ethanol and chloramine T were shown to promote* S. aureus* biofilm formation. This is supported by MS proteomic analysis.

## Figures and Tables

**Table 1:** Tested concentrations of chosen disinfecting agents.

Disinfectant	Characteristic	Range of tested concentrations	Tested concentrations	Concentrations identified as promoting biofilm development (%)
Chloramine T (*µ*g/mL)	Chlorine-releasing agent (CRA)	312–5000	312; 625; 1250; 2500; 5000	Isolates: 1 (B+++): 2500 2 (B++): 2500 3 (B+): 2500
Ethanol (%; v/v)	Alcohol	0.315–20	0.315; 0.63; 1.25; 5; 8; 10; 15; 20	Isolates: 1 (B+++): 0.63; 1.25; 2.5; 5 2 (B++): 0.63; 1.25; 2.5; 5 3 (B+): 1.25

**Table 2:** Differentially regulated proteins in biofilm samples treated with two disinfectants as determined using LC-LTQ/Orbitrap MS.

	Description	PGA	Ethanol (E)			Chloramine T (C)			Differences between disinfectants^b^
			*n*	Median^a^	QD	*n*	Median^a^	QD	
Adherence	Clumping factor A	Q6GB45	23	*3.990* ^*∗∗*^	0.119	45	*3.650* ^*∗∗*^	0.648	
	Clumping factor B	Q6G644	6	*2.358* ^*∗∗*^	0.159	11	0.845	0.316	EC^*∗∗*^
	Elastin-binding protein EbpS	Q6G983	2	0.879	0.164	8	1.259	0.440	
	Enolase	A7WZT2	88	0.998^*∗*^	0.087	83	*1.131* ^*∗∗*^	0.203	
	Extracellular adherence protein Eap	D9RNP1	4	*3.271* ^*∗*^	0.379	6	*2.807* ^*∗∗*^	0.144	
	Fibrinogen-binding protein	P68799	2	1.334	0.109	4	*2.114* ^*∗∗*^	0.059	
	Fibronectin-binding protein A	Q6G6H3	4	0.267^*∗*^	0.079	8	*1.655* ^*∗∗*^	0.125	EC^*∗∗*^
	Immunoglobulin-binding protein A	Q8NYT0	75	0.091^*∗∗*^	0.058	45	0.018^*∗∗*^	0.010	
	Immunoglobulin-binding protein sbi	Q6G6Q3	47	*1.368* ^*∗∗*^	0.263	38	1.088	0.165	EC^*∗*^
	Iron-regulated surface determinant protein A	A7X148	5	*1.358* ^*∗∗*^	0.058	13	0.620^*∗∗*^	0.040	EC^*∗∗*^

Cell wall synthesis and organization	Lipoteichoic acid synthase	Q2FIS2	13	0.786^*∗*^	0.322	15	0.407	0.292	
	N-acetylmuramoyl-L-alanine amidase sle1	Q2FJH7	9	0.885	0.361	4	0.189^*∗∗*^	0.008	EC^*∗*^
	Probable transglycosylase SceD	A7X6T9	2	2.033	0.025	4	0.305^*∗∗*^	0.050	EC^*∗*^

Physiological proteins	Cold shock protein CspA	Q2FH36	7	0.067	0.335	4	0.067^*∗∗*^	0.009	
	Large-conductance mechanosensitive channel	A7X204	6	*1.527* ^*∗∗*^	0.065	4	*1.306* ^*∗*^	0.029	

Transport through membrane	Foldase protein PrsA	A7X3U8	19	*1.287* ^*∗∗*^	0.342	17	0.668^*∗∗*^	0.066	EC^*∗∗*^

Uncharacterized proteins	Uncharacterized lipoprotein MW0073	Q8NYU0	1	1.256	0.000	7	0.852	0.072	
	Uncharacterized lipoprotein SAS2259	Q6G6V2	16	*1.461* ^*∗∗*^	0.177	15	*1.259* ^*∗∗*^	0.106	
	UPF0478 protein SA1560	Q7A531	22	0.862^*∗∗*^	0.102	27	*1.245* ^*∗∗*^	0.125	EC^*∗∗*^

Virulence factors	Penicillin-binding protein 1	Q8NX37	4	*1.247* ^*∗*^	0.041	2	0.323	0.009	
	Phospholipase C	A5IUH1	4	*5.909* ^*∗*^	2.231	2	5.252	0.118	
	Secretory antigen SsaA-like protein	A6QEX4	2	5.578	0.106	0			
	Serine-aspartate repeat-containing protein C	Q6GBS6	32	*2.819* ^*∗∗*^	0.404	12	0.392^*∗∗*^	0.054	EC^*∗∗*^
	Staphylococcal secretory antigen ssaA2	Q2G2J2	22	*4.088* ^*∗∗*^	2.288	21	0.469^*∗∗*^	0.135	EC^*∗∗*^
	Virulence factor EsxA	Q5HJ91	16	*2.002* ^*∗∗*^	0.980	17	*2.261* ^*∗∗*^	1.384	

PGA: protein group accession number; *n*: number of peptides; QD: quartile deviation.

^a^Italic/bold numbers represent statistically significant fold changes in the indicated proteins (upregulated/downregulated) (^*∗∗*^ *P* < 0.01; ^*∗*^ *P* < 0.05; Wilcoxon signed rank test with theoretical median = 1.0).

^b^Statistically significant differences between disinfectants (^*∗∗*^ *P* < 0.01; ^*∗*^ *P* < 0.05; Mann-Whitney test followed by *post hoc* tests).

**Table 3:** Description of proteins listed in Table 2.

Abbreviation	Description
Clumping factor A (clfA)	Cell surface-associated protein implicated in virulence, promotes bacterial attachment exclusively to the gamma-chain of human fibrinogen, induces formation of bacterial clumps (933 aa)
Clumping factor B (clfB)	Cell surface-associated protein implicated in virulence by promoting bacterial attachment to both alpha- and beta-chains of human fibrinogen and inducing the formation of bacterial clumps (913 aa)
Cold shock protein (cspA)	Involved in cold stress response and in the susceptibility to an antimicrobial peptide of human cathepsin G (CG117-136). Regulates yellowish-orange pigment production through a still unclear SigB-dependent mechanism (66 aa)
Elastin-binding protein (ebpS)	Promotes binding of soluble elastin peptides and tropoelastin to *S. aureus* cells although it is not able to promote bacterial adherence to immobilized elastin and, therefore, is not a Microbial Surface Component Recognizing Adhesive Matrix Molecule (MSCRAMM) (486 aa)
Enolase (eno)	Phosphopyruvate hydratase; catalyzes the reversible conversion of 2-phosphoglycerate into phosphoenolpyruvate; it is essential for the degradation of carbohydrates via glycolysis; binds laminin when expressed on the bacterial cell surface; this probably induces destruction of the extracellular matrix, favoring invasion and dissemination (434 aa)
Extracellular adherence protein (eap)	Adherence and invading of eukaryotic cells (985 aa)
Fibrinogen-binding protein (fib)	Binds to host fibrinogen (165 aa)
Fibronectin-binding protein A (fnb)	Promotes bacterial attachment to multiple substrates, such as fibronectin (Fn), fibrinogen (Fg), elastin peptides, and tropoelastin; this confers to *S. aureus* the ability to invade endothelial cells; promotes adherence to and aggregation of activated platelets (1018 aa)
Foldase protein (prsA)	Export protein; plays a major role in protein secretion by helping the posttranslocational extracellular folding of several secreted proteins (320 aa)
IgG-binding protein SBI (sbi)	Interacts with components of both the adaptive and innate host immune system, thereby protecting the cell against the host immune response (436 aa)
Immunoglobulin G-binding protein A (spA)	Function in pathogenesis (508 aa)
Immunoglobulin G-binding protein Sbi	Interacts with components of both the adaptive and innate host immune system, thereby protecting the cell against the host immune response (436 aa)
Immunoglobulin-binding protein (sbi)	Interacts with components of both the adaptive and innate host immune system, thereby protecting the cell against the host immune response (436 aa)
Iron-regulated surface determinant protein A (isdA)	LPXTG cell-wall surface anchor protein; transfers its hemin to hemin-free IsdC (apo-IsdC) directly probably through the activation of the holo-IsdA-apo-IsdC complex and driven by the higher affinity of apo-IsdC for the cofactor; the reaction is reversible; binds transferrin, lactoferrin, heme, hemoglobin, hemin, fetuin, asialofetuin, protein A; also binds fibronectin and chains B, beta and gamma of fibrinogen, promoting clumping of *S. aureus* with fibrinogen; was also shown to adhere to plastic (350 aa)
Large-conductance mechanosensitive channel (mscL)	Channel that opens in response to stretch forces in the membrane lipid bilayer; may participate in the regulation of osmotic pressure changes within the cell (120 aa)
Lipoteichoic acid synthase (ltaS)	Sulfatase; catalyzes the polymerization of lipoteichoic acid (LTA) polyglycerol phosphate, a reaction that presumably uses phosphatidylglycerol (PG) as substrate is required for staphylococcal growth and cell division process (646 aa)
N-acetylmuramoyl-L-alanine amidase (sle1)	Peptidoglycan hydrolase involved in the splitting of the septum during cell division; binds to both alpha- and beta-chains of human fibrinogen as well as fibronectin, which suggests a role in the colonization of host factor-coated material or host tissue; also exhibits lytic activity against *S. carnosus* and *S. aureus* cells but not against *M. luteus* cells (334 aa)
Penicillin-binding protein 1 (pbp1)	Penicillin-binding protein 1 (744 aa)
Phospholipase C (hlb)	Bacterial hemolysins are exotoxins that attack blood cell membranes and cause cell rupture; beta-hemolysin is a phospholipase C with specific activity toward sphingomyelins; has a high specificity for sphingomyelin and hydrolyzes lysophosphatidylcholine at a much lower rate but has no activity toward phosphatidylcholine, phosphatidylethanolamine, or phosphatidylserine (330 aa)
Probable transglycosylase (sceD)	Cleaves peptidoglycan and affects clumping and separation of bacterial cells (231 aa)
Secretory antigen SsA-like protein	Immunogenic protein (267 aa)
Serine-aspartate repeat-containing protein C (sdrC)	sdrC protein; cell surface-associated protein which possibly mediates interactions of *S. aureus* with components of the extracellular matrix of higher eukaryotes; may bind calcium (947 aa)
Staphylococcal secretory antigen Ss aa2 (scaD)	Immunogenic protein (265 aa)
Virulence factor (esxA)	Hypothetical protein; virulence factor that is important for the establishment of infection in the host (97 aa)

source: http://www.string-db.org.

## References

[1] Ray B., Bhunia A. K. (2008). *Fundamental Food Microbiology*.

[2] Gutiérrez D., Delgado S., Vázquez-Sánchez D. (2012). Incidence of staphylococcus aureus and analysis of associated bacterial communities on food industry surfaces. *Applied and Environmental Microbiology*.

[3] Arciola C. R., Campoccia D., Gamberini S., Baldassarri L., Montanaro L. (2005). Prevalence of cna, fnbA and fnbB adhesin genes among *Staphylococcus aureus* isolates from orthopedic infections associated to different types of implant. *FEMS Microbiology Letters*.

[4] Foster T. J., Höök M. (1998). Surface protein adhesins of *Staphylococcus aureus*. *Trends in Microbiology*.

[5] Halliman D. G., Ahearn D. G. (2004). Relative susceptibilities to vancomycin and quinupristin-dalfopristin of adhered and planktonic vancomycin-resistant and vancomycin-susceptible coagulase-negative staphylococci. *Current Microbiology*.

[6] Bridier A., Briandet R., Thomas V., Dubois-Brissonnet F. (2011). Resistance of bacterial biofilms to disinfectants: a review. *Biofouling*.

[7] Cucarella C., Solano C., Valle J., Amorena B., Lasa Í., Penadés J. R. (2001). Bap, a *Staphylococcus aureus* surface protein involved in biofilm formation. *Journal of Bacteriology*.

[8] Latasa C., Solano C., Penadés J. R., Lasa I. (2006). Biofilm-associated proteins. *Comptes Rendus—Biologies*.

[9] Cramton S. E., Gerke C., Schnell N. F., Nichols W. W., Götz F. (1999). The intercellular adhesion (ica) locus is present in Staphylococcus aureus and is required for biofilm formation. *Infection and Immunity*.

[10] Abraham N. M., Jefferson K. K. (2012). *Staphylococcus aureus* clumping factor B mediates biofilm formation in the absence of calcium. *Microbiology*.

[11] Geoghegan J. A., Monk I. R., O'Gara J. P., Foster T. J. (2013). Subdomains N2N3 of fibronectin binding protein a mediate *Staphylococcus aureus* biofilm formation and adherence to fibrinogen using distinct mechanisms. *Journal of Bacteriology*.

[12] Schroeder K., Jularic M., Horsburgh S. M. (2009). Molecular characterization of a novel *Staphylococcus aureus* surface protein (SasC) involved in cell aggregation and biofilm accumulation. *PLoS ONE*.

[13] Geoghegan J. A., Corrigan R. M., Gruszka D. T. (2010). Role of surface protein SasG in biofilm formation by Staphylococcus aureus. *Journal of Bacteriology*.

[14] Merino N., Toledo-Arana A., Vergara-Irigaray M. (2009). Protein A-mediated multicellular behavior in *Staphylococcus aureus*. *Journal of Bacteriology*.

[15] Foster T. J., Geoghegan J. A., Ganesh V. K., Höök M. (2014). Adhesion, invasion and evasion: the many functions of the surface proteins of Staphylococcus aureus. *Nature Reviews Microbiology*.

[16] Patti J. M., Allen B. L., McGavin M. J., Höök M. (1994). MSCRAMM-mediated adherence of microorganisms to host tissues. *Annual Review of Microbiology*.

[17] McAleese F. M., Walsh E. J., Sieprawska M., Potempa J., Foster T. J. (2001). Loss of clumping factor B fibrinogen binding activity by *Staphylococcus aureus* involves cessation of transcription, shedding and cleavage by metalloprotease. *The Journal of Biological Chemistry*.

[18] Bischoff M., Dunman P., Kormanec J. (2004). Microarray-based analysis of the Staphylococcus aureus *σ*
^*B*^ Regulon. *Journal of Bacteriology*.

[19] Chiou R. Y.-Y., Phillips R. D., Zhao P., Doyle M. P., Beuchat L. R. (2004). Ethanol-mediated variations in cellular fatty acid composition and protein profiles of two genotypically different strains of *Escherichia coli*O157:H7. *Applied and Environmental Microbiology*.

[20] Silveira M. G., Baumgärtner M., Rombouts F. M., Abee T. (2004). Effect of adaptation to ethanol on cytoplasmic and membrane protein profiles of *Oenococcus oeni*. *Applied and Environmental Microbiology*.

[21] Fried V. A., Novick A. (1973). Organic solvents as probes for the structure and function of the bacterial membrane: effects of ethanol on the wild Type and an ethanol-resistant mutant of *Escherichia coli* K-12. *Journal of Bacteriology*.

[22] Terracciano J. S., Kashket E. R. (1986). Intracellular conditions required for initiation of solvent production by *Clostridium acetobutylicum*. *Applied and Environmental Microbiology*.

[23] Stief T. W. (2003). The physiology and pharmacology of singlet oxygen. *Medical Hypotheses*.

[24] Rolland S. L., Carrick T. E., Walls A. W., McCabe J. F. (2007). Dentin decontamination using chloramine T prior to experiments involving bacteria. *Dental Materials*.

[25] Bal Krishna K. C., Sathasivan A., Ginige M. P. (2013). Microbial community changes with decaying chloramine residuals in a lab-scale system. *Water Research*.

[26] Rode T. M., Langsrud S., Holck A., Møretrø T. (2007). Different patterns of biofilm formation in *Staphylococcus aureus* under food-related stress conditions. *International Journal of Food Microbiology*.

[27] Tjalsma H., Lambooy L., Hermans P. W., Swinkels D. W. (2008). Shedding & shaving: disclosure of proteomic expressions on a bacterial face. *Proteomics*.

[28] Solis N., Larsen M. R., Cordwell S. J. (2010). Improved accuracy of cell surface shaving proteomics in *Staphylococcus aureus* using a false-positive control. *Proteomics*.

[29] Rodríguez-Ortega M. J., Norais N., Bensi G. (2006). Characterization and identification of vaccine candidate proteins through analysis of the group A *Streptococcus* surface proteome. *Nature Biotechnology*.

[30] Doro F., Liberatori S., Rodríguez-Ortega M. J. (2009). Surfome analysis as a fast track to vaccine discovery: identification of a novel protective antigen for group B Streptococcus hypervirulent strain COH1. *Molecular and Cellular Proteomics*.

[31] Dreisbach A., van der Kooi-Pol M. M., Otto A. (2011). Surface shaving as a versatile tool to profile global interactions between human serum proteins and the *Staphylococcus aureus* cell surface. *Proteomics*.

[32] Ythier M., Resch G., Waridel P. (2012). Proteomic and transcriptomic profiling of *Staphylococcus aureus* surface LPXTG-proteins: correlation with agr genotypes and adherence phenotypes. *Molecular and Cellular Proteomics*.

[33] Stepanović S., Vuković D., Hola V. (2007). Quantification of biofilm in microtiter plates: overview of testing conditions and practical recommendations for assessment of biofilm production by staphylococci. *APMIS*.

[34] Wiśniewski J. R., Zougman A., Nagaraj N., Mann M. (2009). Universal sample preparation method for proteome analysis. *Nature Methods*.

[35] Boersema P. J., Raijmakers R., Lemeer S., Mohammed S., Heck A. J. R. (2009). Multiplex peptide stable isotope dimethyl labeling for quantitative proteomics. *Nature Protocols*.

[36] Tashiro Y., Inagaki A., Ono K. (2014). Low concentrations of ethanol stimulate biofilm and pellicle formation in Pseudomonas aeruginosa. *Bioscience, Biotechnology and Biochemistry*.

[37] Houari A., Di Martino P. (2007). Effect of chlorhexidine and benzalkonium chloride on bacterial biofilm formation. *Letters in Applied Microbiology*.

[38] Korem M., Gov Y., Rosenberg M. (2010). Global gene expression in *Staphylococcus aureus* following exposure to alcohol. *Microbial Pathogenesis*.

[39] Redelman C. V., Maduakolam C., Anderson G. G. (2012). Alcohol treatment enhances *Staphylococcus aureus* biofilm development. *FEMS Immunology & Medical Microbiology*.

[40] Di Ciccio P., Vergara A., Festino A. R. (2015). Biofilm formation by *Staphylococcus aureus* on food contact surfaces: relationship with temperature and cell surface hydrophobicity. *Food Control*.

[41] da Silva Meira Q. G., de Medeiros Barbosa I., Alves Aguiar Athayde A. J., de Siqueira-Júnior J. P., de Souza E. L. (2012). Influence of temperature and surface kind on biofilm formation by *Staphylococcus aureus* from food-contact surfaces and sensitivity to sanitizers. *Food Control*.

[42] Zmantar T., Kouidhi B., Miladi H., Mahdouani K., Bakhrouf A. (2010). A microtiter plate assay for *Staphylococcus aureus* biofilm quantification at various pH levels and hydrogen peroxide supplementation. *New Microbiologica*.

[43] Post D. M. B., Held J. M., Ketterer M. R. (2014). Comparative analyses of proteins from Haemophilus influenzae biofilm and planktonic populations using metabolic labeling and mass spectrometry. *BMC Microbiology*.

[44] Resch A., Leicht S., Saric M. (2006). Comparative proteome analysis of *Staphylococcus aureus* biofilm and planktonic cells and correlation with transcriptome profiling. *Proteomics*.

[45] Resch A., Rosenstein R., Nerz C., Götz F. (2005). Differential gene expression profiling of Staphylococcus aureus cultivated under biofilm and planktonic conditions. *Applied and Environmental Microbiology*.

[46] Arrecubieta C., Matsunaga I., Asai T., Naka Y., Deng M. C., Lowy F. D. (2008). Vaccination with clumping factor A and fibronectin binding protein A to prevent Staphylococcus aureus infection of an aortic patch in mice. *Journal of Infectious Diseases*.

[47] Carneiro C. R. W., Postol E., Nomizo R., Reis L. F. L., Brentani R. R. (2004). Identification of enolase as a laminin-binding protein on the surface of *Staphylococcus aureus*. *Microbes and Infection*.

[48] Seyer D., Cosette P., Siroy A. (2005). Proteomic comparison of outer membrane protein patterns of sessile and planktonic *Pseudomonas aeruginosa* cells. *Biofilms*.

[49] Wang Y., Yi L., Wu Z. (2012). Comparative proteomic analysis of streptococcus suis biofilms and planktonic cells that identified biofilm infection-related immunogenic proteins. *PLoS ONE*.

[50] Jones R. C., Deck J., Edmondson R. D., Hart M. E. (2008). Relative quantitative comparisons of the extracellular protein profiles of *Staphylococcus aureus* UAMS-1 and its sarA, agr, and sarA agr regulatory mutants using one-dimensional polyacrylamide gel electrophoresis and nanocapillary liquid chromatography coupled with tandem mass spectrometry. *Journal of Bacteriology*.

[51] Becher D., Hempel K., Sievers S. (2009). A proteomic view of an important human pathogen—towards the quantification of the entire *Staphylococcus aureus* proteome. *PLoS ONE*.

[52] Curreem S. O. T., Watt R. M., Lau S. K. P., Woo P. C. Y. (2012). Two-dimensional gel electrophoresis in bacterial proteomics. *Protein and Cell*.

[53] Islam N., Kim Y., Ross J. M., Marten M. R. (2014). Proteomic analysis of Staphylococcus aureus biofilm cells grown under physiologically relevant fluid shear stress conditions. *Proteome Science*.

[54] Mohamed N., Visai L., Speziale P., Ross J. M. (2000). Quantification of Staphylococcus aureus cell surface adhesins using flow cytometry. *Microbial Pathogenesis*.

[55] Grandi G. (2010). Bacterial surface proteins and vaccines. *F1000 Biology Reports*.

[56] Bøhle L. A., Riaz T., Egge-Jacobsen W. (2011). Identification of surface proteins in Enterococcus faecalis V583. *BMC Genomics*.

